# IQ-CPR Meter for Chest Compression Monitoring During Simulated Cardiopulmonary Resuscitation; a Comparative Study

**Published:** 2020-09-19

**Authors:** Phatthranit Phattharapornjaroen, Suwitchaya Surapornpaiboon, Phanorn Chalermdamrichai, Yuwares Sittichanbuncha, Kittisak Sawanyawisuth

**Affiliations:** 1 Department of Emergency Medicine, Faculty of Medicine Ramathibodi Hospital, Mahidol University, Bangkok, Thailand.; 2 Department of Medicine, Faculty of Medicine, Sleep Apnea Research Group, Khon Kaen University, Khon Kaen, Thailand.

**Keywords:** Quality indicators, health care, heart arrest, cardiopulmonary resuscitation, simulation training

## Abstract

**Introduction:**

Adequate chest compression is crucial for cardiopulmonary resuscitation (CPR). There are several chest compression monitoring devices with different costs. This study aimed to evaluate the agreement rate of Improved Quality of Cardiopulmonary Resuscitation meter (IQ-CPR meter) and automated external defibrillator (AED) in chest compression quality monitoring.

**Methods:**

In this comparative study, participants were instructed to perform chest compression on the CPR manikins with the set rate of 110 times/minute for two minutes. The CPR manikins had two monitors: AED (R series^®^, Zoll company) and IQ-CPR meter. AED showed the depth and speed of chest compression on the screen, while IQ-CPR meter showed the depth of each chest compression by color light for quality of chest compression depth. Video-based analysis was used to compare the chest compression quality monitoring between the 2 devices.

**Results:**

There were 27 participants in the study with a mean age and body mass index (standard deviation; SD) of 26.00 (5.65) years, and 22.93 (3.62) kg/m^2^ (70.37% male). The median (1^st^ to 3^rd^ quartile range) of chest compression experience was 3 (1.00-6.50) years. The mean (SD) of chest compression rate was 107 (5.29) times/minute. Based on Cohen’s Kappa correlation, agreement between the IQ-CPR meter and the AED was 66.54%.

**Conclusion:**

The IQ-CPR meter had fair agreement with the computerized chest compression monitoring device with lower cost and simple, real time audiovisual feedback.

## Introduction

Cardiac arrest is a fatal condition affecting more than 290,000 adult patients each year in the United States (1). Over 50% of cardiac arrests are caused by cardiac causes; with the survival rate of approximately 20%. Chest compression is an important part of cardiopulmonary resuscitation (CPR) that should be delivered to the victim as soon as possible (2). The 2017 version of American Heart Association (AHA) recommendations recommends chest compression-only CPR for lay rescuers trained in chest compression. The recommended chest compression: ventilation ratio is 30:2.

Since 81% of cardiac arrest patients had non-shockable rhythm at presentation (1), chest compression is very crucial. The 2015 version of AHA recommendations recommended a chest compression rate between 100 and 120 times/minute with depth between 5 and 6 cm (3). To comply with these recommendations, the quality of chest compressions needed to be monitored. There are several devices to assist chest compression such as automated feedback or audiovisual feedback (4-6). The prototype defibrillator with feedback improved adequate chest compression depth from 24% to 53% as well as short term survival (5). However, some devices may have high prices, which may not be suitable for the resource-limited health care facilities. 

**Figure 1:**
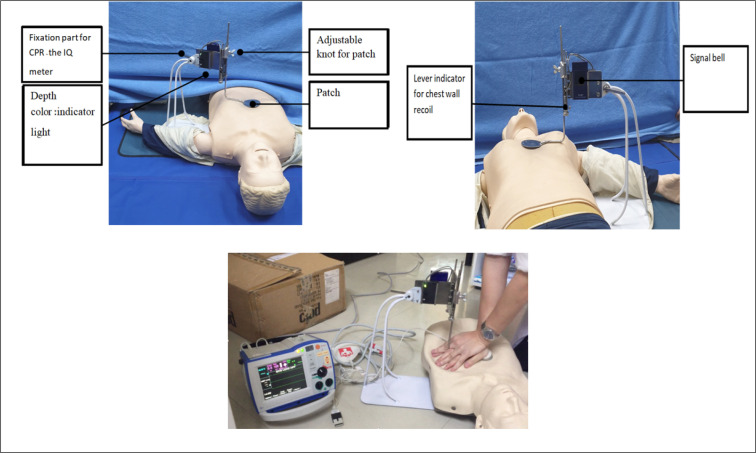
Improved Quality of Cardiopulmonary Resuscitation meter (IQ-CPR meter) components

The Improved Quality of Cardiopulmonary Resuscitation meter (IQ-CPR meter) is a low-cost device with the aim of improving the quality of CPR, particularly chest compression. This custom-made device produced by the authors, costs 83 USD and provides real time feedback for chest compression. The IQ-CPR meter has three main components: adjustable patch, signal bell, and depth indicator (Figure 1). The adjustable patch is placed on the chest of the victim to evaluate chest compression depth. The signal bell is set for 110 times/minute to lead constant and proper chest compression rate by a ding sound. The depth indicator shows real-time feedback for adequate chest compression depth: no light indicates shallow depth (< 5 cm), green light indicates good depth (5-6 cm), and red light indicates too much depth (> 6 cm). This study aimed to evaluate if the IQ-CPR meter shows agreement with a costly chest compression monitoring device.

## Methods

**Study design and setting**

This comparative study was conducted between the newly invented IQ-CPR meter and the standard CPR monitoring device. We conducted the study at Department of Emergency Medicine, Ramathibodi Hospital, Mahidol University, Bangkok, Thailand. The study period was between May 1st and September 30th, 2018. Baseline characteristics and chest compression experiences of eligible patients were recorded. The study protocol was approved by the committee on human rights related to research involving human subjects, Mahidol University (MURA2561/86). The participants gave informed consent prior to participation in the study.

**Participants**

The inclusion criteria were being an adult aged between 18 and 60 years, being among the active medical personnel at the ED, and willing to participate in the study. Those who were pregnant or had comorbid diseases, which could cause inability to perform chest compression, such as heart disease or anemia were excluded. There were four categories of medical personnel participating in the study: faculty members or residents of emergency department (ED), 5th/6th year medical students, paramedic students, and emergency medical services (EMS) staff. 

**Procedure**

We instructed the participants to perform chest compression on the CPR manikins with the set rate of 110 times/minute; for two minutes. The CPR manikins had two monitors: automated external defibrillator (AED) R series®, Zoll company and IQ-CPR meter (Figures 1). AED showed the depth and speed of chest compression rate on the screen, while the IQ-CPR meter showed the depth of each chest compression by color light. If the chest compression had a depth less than 5 cm, 5-6 cm, and more than 6 cm, the lights of the IQ-CPR showed no light, green, and red, respectively. The participants performed chest compression for two minutes with video recording by the Sony HDR PJ440. Data from the AED and the IQ-CPR, including rate, depth, and full recoil of chest compression for each participant were retrieved from the video. The main primary outcome was chest compression depth, while the secondary outcomes included chest compression rate, and full recoil of chest compression. The data from AED were used as the standard values: chest compression depth, chest compression rate, and full recoil of chest compression. Data from the IQ-CPR were compared with the standard values from the AED. Chest compression depth of each participant was scored by two authors (PP and SS) watching the video. The interrater reliability was 95%.

**Statistical** **analysis**

Due to no previous data on the correlation of the IQ-CPR meter and the standard monitoring, we performed an example study on the correlation between both devices on depth of chest compression. The agreement rate between the 2 devices on chest compression for two minutes was 0.85. With an error (d) of 0.02, alpha of 0.05, and Z (0.975) or 1.959964, the required sample size was 6,123 times of chest compression. The required sample size was 27 participants to perform chest compression for two minutes with the rate of 110 times/minute. Descriptive statistics were used to report baseline characteristics and outcome of the participants. Baseline characteristics were age, sex, experience of chest compression, and body mass index, while the outcome of chest compression was depth, rate, and full recoil of chest compression. Data were presented as mean (standard deviation; SD) or median (1st to 3rd quartile range) for numerical variables based on normal distribution pattern. For categorical data, number (percentage) was reported. The main primary outcome was good depth of chest compression: 5-6 cm depth. The correlation between good depth of chest compression reported by the AED and the IQ-CPR meter was evaluated using Cohen’s Kappa coefficient. There were three levels of agreement based on Cohen’s Kappa coefficient: excellent agreement (> 0.75), intermediate to good agreement (0.40-0.75), and poor agreement (<0.40). A subgroup analysis of various baseline characteristics was performed using Cohen’s Kappa coefficient. Sensitivity and specificity of each category of the IQ-CPR meter were calculated. The statistical analyses were performed using STATA software, version 10.1 (College Station, Texas, USA).

## Results

**Baseline characteristics of participants**

There were 27 participants in the study, categorized as faculty members or residents in the ED (7 participants), clinical year medical students (7 participants), paramedic students (7 participants), and EMS staff (6 participants). The average age and body mass index (SD) of the participants were 26.00 (5.65) years and 22.93 (3.62) kg/m^2^. 70.37% (19 participants) were male. The median (1st to 3rd quartile range) of chest compression experience was 3 (1.00-6.50) years.

The mean (SD) of chest compression rate was 107 (5.29) times/minute (100 -123). The majority of participants had a chest compression rate between 100-120 times/minute; 25 participants (92.59%). The other two participants had chest compression rate of 121 and 123 times/minute (7.41%). The median (1st to 3rd quartile range) of full recoil rate was 15% (3.00-39.50). 

**Agreement rate**

Cohen’s Kappa coefficient between the IQ-CPR meter and the AED was 67.25% (Table 1). The subgroup analysis on baseline characteristics showed that Cohen’s Kappa coefficients ranged from 51.54% to 80.39%: the highest in paramedic students. There were 5,954 chest compressions attempts in the study. The IQ-CPR had the highest sensitivity in the green light category (84.54%) and the highest specificity in the red light category (99.96%) as shown in Table 2. 

**Table 1:** Cohen's Kappa coefficients for chest compression depth between AED and IQ-CPR meter in various populations

**Study population**	**Cohen's Kappa coefficient** ** (** **95** **%** ** CI** **)**
All patients	67.25% (66.02%-68.46%)
Male	65.63% (64.15%-67.08%)
Female	71.21% (68.98%-73.38%)
EP/residents	71.20% (68.84%-73.48%)
Medical students, clinical years	63.15% (60.62%-65.62%)
Paramedic students	80.39% (78.31%-82.36%)
EMS personnel	51.54% (48.75%-54.32%)
Chest compression experience < 3 years	71.92% (70.27%-73.52%)
Chest compression experience > 3 years	62.24% (60.41%-64.05%)

CI: confidence interval; EP: electrophysiologist; EMS: emergency medical services; AED: automated external defibrillator; IQ-CPR meter: Improved Quality of Cardiopulmonary Resuscitation meter.

**Table 2:** Sensitivity and specificity of the IQ-CPR meter compared with AED for chest compression depth

**Category**	**Sensitivity** ** (** **95** **%** ** CI** **)**	**Specificity** ** (** **95** **%** ** CI** **)**
No light (depth < 5 cm)	32.23 (30.30-34.21)	84.82 (83.62-85.96)
Green light (depth 5-6 cm)	84.54 (83.32-85.70)	31.35 (29.55-33.37)
Red light (depth > 6 cm)	1.26 (0.03-6.85)	99.96 (99.88-100.00)

CI: confidence interval; AED: automated external defibrillator; IQ-CPR meter: Improved Quality of Cardiopulmonary Resuscitation meter

## Discussion

The IQ-CPR meter had fair agreement with the AED device with a Cohen’s Kappa coefficient of 67.25%. Both devices provide real-time feedback on chest compression during CPR. The advantages of IQ-CPR meter over the R series^®^ AED include lower cost (83 USD vs 13,333 USD), and being lighter. Additionally, the chest compression can be conducted by the bell sound, leading to constant and correct chest compression rate (mean 107 times/minute and 95.59% appropriate rate). For the compression depth, the light indicator indicates real-time compression depth. In contrast, the participants needed to look at the R series^®^ AED screen and interpret if the depth was adequate. As previously reported, a simple light device improved percentage target depth from 48.86% to 72.95%; p 0.036 compared to times when light device was not used (7). The same study also showed that light can improve target rate from 35.82% to 67.09%; p 0.024. This study showed that a simple sound could also lead to adequate chest compression rate as well. The IQ-CPR meter uses simple light and sound, real-time audio-visual signals, to lead the participants to perform adequate chest compression in terms of rate and depth. Similarly, previous studies showed that real-time audio-visual feedback is crucial and can improve chest compression quality (5, 8, 9). 

The IQ-CPR meter had a high sensitivity (84.54%) for good chest compression depth identified by the green light. Therefore, the green light signal by the IQ-CPR meter may be a good assistant for chest compression during CPR (Table 2). Regarding the subgroup analyses, all Cohen’s Kappa coefficients were all indicative of good agreement (Table 1). Note that the paramedic students had a slightly higher coefficient (80.39%). A previous study found that paramedic students may perform better quality CPR compared with medical students (10). The paramedic students had a significantly higher rate of appropriate chest compression depth than medical students in clinical years (50.7 vs 47.3 mm; p 0.01). However, these findings may or may not be related to the highest Cohen’s Kappa coefficient found in this study. 

Another advantage of the IQ-CPR meter is that it can show full recoil of the chest wall through the lever connected to the patch (Figure 1). If the chest wall is fully recoiled, the lever will be at the original position. However, it might be slightly difficult to look at the lever and light at the same time during chest compression, resulting in low rate of full recoil in this study (15%). However, there is no evidence of full recoil rate and survival of cardiac arrest patients. 

There are other cheaper chest compression monitoring devices such as mobile phone or smart watch. A previous study found that smart phone and smart watch had comparable chest compression quality with the manikin system (11, 12). Note that audio feedback was given to guide the performer to a compression rate between 100-120 times/minute. For the IQ-CPR meter, no audio feedback is needed with the compression rate accuracy of 95.59%. 

The main strength of this study is the IQ-CPR meter, which is cheap and can be a helpful tool for adequate chest compression quality. Further studies with real patients may be needed to confirm the results of this study. 

## Limitations

The main limitation of the IQ-CPR meter is that it requires a flat surface for device setup. And, it may not be suitable for children. Low body weight may cause instability in the IQ-CPR base. Finally, there might be some degrees of disagreement between raters, because chest compression depths were defined obviously and clearly by the AED and the IQ-CPR. The AED showed compression depth in cm on monitor, while the IQ-CPR showed chest compression depth by light (no light, green, and red). Therefore, the magnitude of bias is low (95% agreement). 

## Conclusion

The IQ-CPR meter had fair agreement with the computerized chest compression monitoring device, with lower cost and simple, real-time audio-visual feedback.

## Authors’ contribution

PP designed the study, analyzed data, interpreted data, and drafted a manuscript; SS collected and interpreted data; PC invented the IQ-CPR and interpreted data; YS interpreted data and supervised the research; KS involved in data analysis and drafted a manuscript. All authors read and approved the final manuscript. 

## Funding/Support

This research did not receive any specific grant from funding agencies in the public, commercial, or not-for-profit sectors.

## Conflict of interest

The authors report no conflicts of interest
